# Nationwide Survey on Bariatric and Metabolic Surgery in Korea: 2003–2013 Results

**DOI:** 10.1007/s11695-015-2030-y

**Published:** 2015-12-29

**Authors:** Hyuk-Joon Lee, Hye Seong Ahn, Youn Baik Choi, Sang-Moon Han, Sang-Uk Han, Yoon-Seok Heo, Kyoung Yul Hur, Eung Kook Kim, Ji Hun Kim, Young-Jin Kim, Hong Chan Lee, Joo Ho Lee, Do-Joong Park, Yun-Chan Park, Seung Ho Choi, The Information Committee of the Korean Society of Bariatric and Metabolic Surgery

**Affiliations:** Department of Surgery, Seoul National University Hospital, Seoul, Korea; Department of Surgery, Seoul National University-SMG Boramae Medical Center, Seoul, Korea; Department of Surgery, Asan Medical Center, Seoul, Korea; Department of Surgery, CHA Gangnam Medical Center, Seoul, Korea; Department of Surgery, Ajou University Hospital, Suwon, Korea; Department of Surgery, Inha University Hospital, Incheon, Korea; Department of Surgery, SoonChunHyang University Seoul Hospital, Seoul, Korea; Department of Surgery, Yeouido St. Mary’s Hospital, Catholic University of Korea, Seoul, Korea; Wellness Hospital, Busan, Korea; Chan BariART Clinic, Seoul, Korea; Department of Surgery, Ewha Medical Center, Seoul, Korea; Department of Surgery, Seoul National University Bundang Hospital, Seongnam, Korea; Seoul Slim Surgery, Seoul, Korea; Department of Surgery, Gangnam Severance Hospital, Yonsei University College of Medicine, 211 Engjuro (146-92 Dongok-dong), Gangnam-gu, Seoul, 135-720 Korea

**Keywords:** Nationwide survey, Bariatric surgery, Metabolic surgery, Complication, Korea

## Abstract

A survey to evaluate the current status of bariatric and metabolic operations in Korea was conducted. Data from 5467 cases (32 hospitals) were collected. The annual numbers of bariatric and metabolic operations increased each year, from 139 in 2003 to 1686 in 2013. Adjustable gastric band (AGB, 67.2 %) was the most common operation, followed by sleeve gastrectomy (SG, 14.2 %), and Roux-en-Y gastric bypass (RYGB, 12.7 %). Mean patient age and body mass index (BMI) were 35.4 years and 35.9 kg/m2, respectively. In-hospital morbidity and mortality rates were 6 % (114/2305) and 0.25 % (5/2176), respectively. In Korea, AGB was the most common operation because of the availability and activity of specialized bariatric clinics. These national survey results established a baseline for future data collection.

## Introduction

The 2007 Korea National Health and Nutrition Examination Survey results indicated that the proportion of obese people (body mass index [BMI] ≥30 kg/m^2^) had increased to 4.1 % of the Korean population [1]. This increase in the prevalence of obesity has become a serious public health concern. Bariatric operations have low morbidity and mortality rates and reduce metabolic syndrome and obesity related comorbidities [2–5]. They increased in number after their introduction in Korea. Several studies from one institution and reports from the National Evidence-based Collaborating Agency were published. However, the current status of bariatric and metabolic surgery in Korea was rarely reported until now.

A retrospective nationwide survey was conducted to evaluate the current status of bariatric and metabolic operation and to evaluate short- and long-term outcomes by the Korean Society of Bariatric and Metabolic Surgery. This survey was needed to answer the annual survey of International Federation for the Surgery of Obesity and Metabolic Disorders (IFSO).

## Patients and Method

The nationwide survey was announced at the spring meeting of the Korean Society of Bariatric and Metabolic Surgery on 12 April 2014. The society’s information committee prepared and provided the Microsoft Excel sheet format to all members of the society and collected data via e-mail after the participating institutions got approval of this study from the Institutional Review Board (H-1404-104-572). We collected data on the first case of each institution, and on subsequent cases undergoing bariatric or metabolic surgery, till 2013. Patients who underwent resection of gastric cancer or other gastric pathology with bariatric or metabolic surgery were excluded from the study. The database included patient characteristics (age, sex, height, weight, comorbidities, and the American Society of Anesthesiology score), operation characteristics (operation type, revision surgery, minimally invasiveness, and operation time (min)), and postoperative morbidities and mortality.

The data were analyzed using SPSS® version 20.0 software (IBM, Armonk, New York, USA). The *χ*2 test, Fisher’s exact test, and Student’s *t* test were used for statistical analysis. A *p* value <0.05 (two-sided) was regarded as statistically significant result in all analyses.

## Results

### Data Acquisition

From 23 April 2014 to 3 Nov 2014, 32 institutions (27 university hospital and 5 bariatric centers) sent data on 5436 cases. Twenty-eight institutions reported the patient and operation characteristics of 3428 cases. Four institutions reported only the type of operations on 2004 cases. And 25 institutions reported the postoperative morbidities and mortality of 1656 cases.

### Annual Number and Types of Metabolic and Bariatric Surgery

**Fig. 1:**
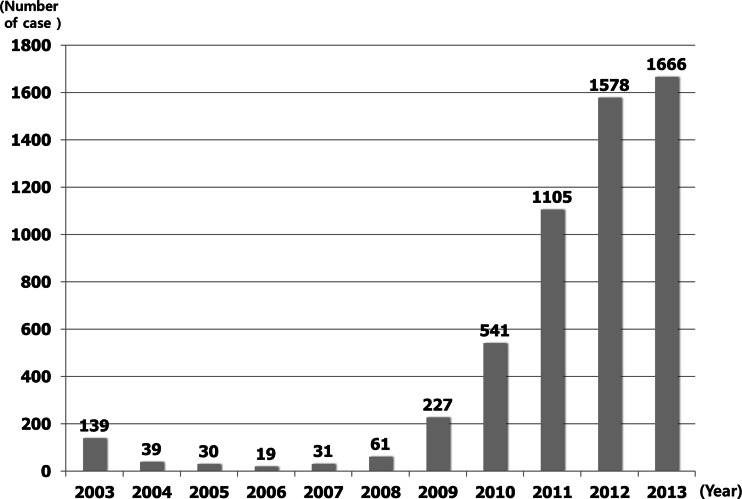
Annual numbers of bariatric and metabolic surgery

The first patients were operated in 2003. After 139 cases underwent bariatric or metabolic operations in 2003, <100 cases underwent operations annually from 2004 to 2008. The annual case numbers of bariatric and metabolic surgery sharply increased from 2009 (227 in 2009, 541 in 2010, and 1666 in 2013) (Fig. 1).

The most frequent operation was adjustable gastric band (AGB, *n* = 3676 (67.2 %)), followed by sleeve gastrectomy (SG, *n* = 775 (14.2 %)), Roux-en-Y gastric bypass (RYGB, *n* = 697 (12.8 %)), and mini gastric bypass (MGB, *n* = 182 (3.3 %)). Duodeno-jejunal bypass (DJB, *n* = 52, 1.0 %), resectional gastric bypass (*n* = 25, 0.5 %), gastric plication (*n* = 10, 0.2 %), biliopancreatic diversion (*n* = 1), and biliopancreatic diversion with duodenal switch diversion (*n* = 1) were also performed.

**Fig. 2:**
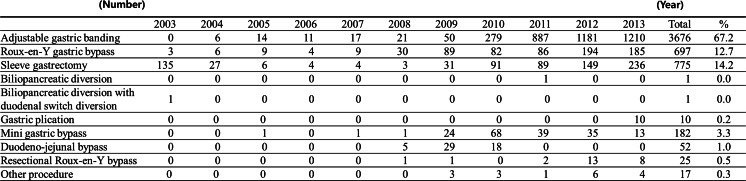
Annual numbers of bariatric and metabolic surgery according operation types

The annual numbers of AGB rapidly increased after 2009 and reached 1210 cases in 2013; RYGB also increased after 2009, to 193 in 2012. The trend in SG was similar and a total of 236 SGs were performed in 2013 (Fig. 2).

The revision or secondary operation accounted for 2.6 % (*n* = 90) of cases with data on primary or revision operation (*N* = 3460). Among them, most frequent operation was RYGB (*n* = 29), followed by AGB (*n* = 18), and SG (*n* = 12).

### Patients Characteristics with Primary Operation

The mean ± standard deviation (SD) patient age and BMI were 35.4 ± 10.6 years and 35.9 ± 7.4 kg/m2, respectively. The male:female ratio was 1:3.5, and 66.1 % of 3332 patients had comorbidities. The most common comorbidity was fatty liver (36.8 %), followed by diabetes (24.0 %), hypertension (22.4 %), and dyslipidemia (20.8 %).

The proportions of patients with a BMI ≥30, ≥35, or ≥40 kg/m^2^ were 80, 53, and 25 %, respectively. For patients with a BMI ≥ 40 kg/m^2^, the most frequently performed operation was AGB (47.4 %), followed by RYGB (26.0 %), and SG (24.7 %). But, the most common operation for the patients with a BMI < 30 were different (e.g., AGB [54.0 %], MGB [25.1 %], DJB [8.1 %], and RYGB [7.6 %]) from that for the patients with a BMI ≥ 30. The operations for patients with diabetes included RYGB (31.8 %), MGB (20.9 %), SG (19.6 %), AGB (19.1 %) and DJB (6.5 %). AGB was the most common operation for non-diabetic patients (65.9 %).

### Institutions of Metabolic and Bariatric Surgery

The numbers of institutions which performed metabolic and bariatric surgery increased annually, from 4 institutions in 2003 to 14 institutions from 2009 to 29 institutions in 2013. The year 2009 was the first year that an institution performed ≥100 metabolic and bariatric surgeries, and the numbers of these institutions increased to 5 in 2012. The number of institutions which annually performed <10 metabolic and bariatric surgeries per year increased to 19 in 2013.

The first bariatric operation was performed at a university hospital, and until 2008, only university hospitals performed bariatric and metabolic operations. Private bariatric centers were opened in 2009. In 2011, the numbers of bariatric and metabolic surgeries by the private hospitals (*n* = 881) exceeded the numbers of operations performed by the university hospitals (*n* = 224). In 2013, operations were more frequently performed at the private hospitals compared with the university hospitals (1285 vs. 381, respectively).

The patients’ characteristics of private hospitals and university hospital were shown in Table 1. The university hospital patients had higher BMIs and more comorbidities (especially diabetes mellitus), which was reflected in the selection of operation types. RYGB (38.1 %) was the most common surgery performed at the university hospitals, followed by the SG (34.2 %) and AGB (11.1 %). In the university hospitals, MGB (10.1 %), DJB (2.9 %) and resectional gastric bypass (1.4 %) were also performed to treat patients with metabolic syndromes. The private hospitals mainly performed AGB (94.7 %).

**Table 1:** Patients’ characteristics according to institution

Patients	University hospital		Private hospital		*p*
	Case no.		Case no.		
	Mean ± SD	*N* (%) or	Mean ± SD	*N* (%) or	
Age	1719	37.3 ± 11.7	1709	33.5 ± 8.9	0.000
Sex	1722	1: 2.3	1709	1: 6.2	0.000
BMI	1722	36.6 ± 8.0	1708	35.1 ± 6.6	0.000
ASA	813	267:506:40	182	51:131:0	0.002
No. of comorbidities	814	2.2 ± 1.4	1709	1.1 ± 1.4	0.000
No. of patients with comorbidities	814	760 (93.4 %)	1709	869 (50.8 %)	0.000
Diabetes mellitus	814	489 (60.1 %)	1709	128 (7.5 %)	0.000
Preop HbA1C	500	8.0 ± 2.0	773	6.0 ± 1.1	0.000
dyslipidemia	814	120 (14.7 %)	1709	302 (17.7 %)	0.065
Preop cholesterol	628	191.5 ± 40.2	14	202.7 ± 37.8	0.000

### Characteristics of Primary Operation and Postoperative Morbidity and Mortality

Most operations (98.5 %) were performed laparoscopically; only 1.2 % were performed after laparotomy. Nine cases underwent surgeries using a robot. The mean operation time was 96.8 min (Range 30 ∼ 720) and the mean hospital stay was 4.6 days (range 1 ∼ 386).

The rates of in-hospital morbidity and mortality were 6 % (114/2305) and 0.25 % (5/2176), respectively. Most common postoperative morbidity was wound complication (1.4 %, Table 2). Four patients died from pneumonia and respiratory arrest and one patient died from disseminated intravascular coagulation associated with massive hemorrhage. The morbidity rates after RYGB, SG, and AGB were 8, 7, and 1 %, respectively.

**Table 2:** The postoperative morbidities of patients with primary operation

*N* = 2305*	*N*	%
Wound complication	32	1.4
Intra-abdominal bleeding	19	0.8
Intra-luminal bleeding	16	0.7
Pulmonary complication	11	0.5
Postoperative intestinal obstruction/ileus	8	0.3
Anastomosis leak	8	0.3
Anastomosis stenosis	4	0.2
Fluid collection/abscess	4	0.2
Urinary complication	2	0.1
Renal complication	2	0.1
Endocrine complication	1	0.03
Others	16	0.7

*Among 3371 patients with primary operation, the postoperative morbidities data on 2305 cases were collected.

## Discussion

In Korea, the status of bariatric and metabolic procedures was poorly understood because they were not covered by the national health insurance. Therefore, a nationwide survey was performed by the Korean Society of Bariatric and Metabolic Surgery. According to this, the first bariatric operation in Korea was performed in 2003. After the foundation of the Korean Society of Bariatric and Metabolic Surgery in 2008, the annual numbers of metabolic and bariatric operations sharply increased (228 cases in 2009 and 1686 cases in 2013).

According to the IFSO’s worldwide survey results, RYGB was the most common operation from 2003 onward, and use of AGB continuously decreased after 2008 [6]. However, AGB was the most common operation in Korea and it accounted for >70 % of the operation performed between 2011 and 2013. The reason for this difference may be that the ease, speed, reversibility, and no anastomosis in AGB meant that private hospitals were more likely to perform AGB. The increase in AGB was also likely positively affected by the fact that far greater numbers of AGB were performed in private hospitals (e.g., more than triple in 2013) compared with the university hospitals. Although RYGB accounted for >45 % of the operations performed worldwide form 2003 onward, it comprised 11 % of the operations in 2013 in Korea. Resectional gastric bypass accounted for a proportion of bariatric and metabolic surgery in Korea because of the high incidence of gastric cancer and greater confidence in, and experience with, performance of laparoscopic total gastrectomy.

The mean age of the Korean patients was younger compared with the mean ages found by Buchwald’s meta-analysis published in 2004 (39.0 years), Singaporean nationwide survey (40.3 years) and German nationwide survey (≥40.4 years) [2, 7, 8]. The mean BMI of Korean patients was lower than those of three reports, because we included the metabolic operations that were performed in lean patients with type 2 diabetes. In Korea, the patients with a BMI < 30 underwent MGB, and DJB more frequently than the patients with a BMI ≥ 30. The patients with diabetes underwent RYGB, MGB, SG, and DJB more frequently than the patients without diabetes. These trends were affected by the patient selection, which was performed according to the consensus in Asia-Pacific perspective [9, 10]. The introduction and clinical trials of the operations that improve metabolic conditions, such as MGB and DJB, were also related to the patient selection.

The rates of mortality and morbidity were comparable to previously reported rates [2, 7, 8]. The low morbidity rates might have been related to the skill of the bariatric surgeon. Korean bariatric surgeons have extensive experience in performing laparoscopic gastrointestinal surgery due to high incidence of gastric cancer. The low morbidity rate could have also been affected by the collection of in-hospital morbidity data, rather than a 30-day morbidity data. The shorter hospital stays could have resulted in relatively lower morbidity rates.

## Conclusion

We used a national survey to evaluate the status of metabolic and bariatric surgery in Korea and to report Korean data to 2013 survey of IFSO. The numbers of SG and RYGB in Korea increased similarly to worldwide trends, but AGB had the greatest increases and was the most common bariatric and metabolic surgery. All bariatric and metabolic surgeries could be considered to be safely performed. More follow-up data are needed to evaluate efficacy. These results can be used as baseline data for insurance coverage of metabolic and bariatric surgery and to develop the Korean guidelines and quality control standards. The results can also be used to develop future multicenter collaborative studies.
